# Susceptibility of *Anopheles gambiae* to insecticides used for malaria vector control in Rwanda

**DOI:** 10.1186/s12936-016-1618-6

**Published:** 2016-12-01

**Authors:** Emmanuel Hakizimana, Corine Karema, Dunia Munyakanage, Gad Iranzi, John Githure, Jon Eric Tongren, Willem Takken, Agnes Binagwaho, Constantianus J. M. Koenraadt

**Affiliations:** 1 Malaria and Other Parasitic Diseases Division (MOPDD), Rwanda Biomedical Centre, Ministry of Health, Kigali, Rwanda; 2 Laboratory of Entomology, Wageningen University and Research, PO Box 16, 6700 AA Wageningen, The Netherlands; 3 Swiss Tropical and Public Health Institute, Basel, Switzerland; 4 University of Basel, Basel, Switzerland; 5 Integrated Vector Management Project, MOPDD, Abt Associates Inc., Kigali, Rwanda; 6 USAID/PMI Office, Kigali, Rwanda; 7 Department of Global Health and Social Medicine, Harvard Medical School, Boston, MA USA; 8 Geisel School of Medicine, Dartmouth College, Hanover, NH USA; 9 University of Global Health Equity, Kigali, Rwanda

**Keywords:** Insecticide resistance, Rwanda, *Anopheles gambiae*, Vector control, Pyrethroids, Bendiocarb, DDT, Fenitrothion, *kdr* Mutation

## Abstract

**Background:**

The widespread emergence of resistance to pyrethroids is a major threat to the gains made in malaria control. To monitor the presence and possible emergence of resistance against a variety of insecticides used for malaria control in Rwanda, nationwide insecticide resistance surveys were conducted in 2011 and 2013.

**Methods:**

Larvae of *Anopheles gambiae sensu lato* mosquitoes were collected in 12 sentinel sites throughout Rwanda. These were reared to adults and analysed for knock-down and mortality using WHO insecticide test papers with standard diagnostic doses of the recommended insecticides. A sub-sample of tested specimens was analysed for the presence of knockdown resistance (*kdr*) mutations.

**Results:**

A total of 14,311 mosquitoes were tested and from a sample of 1406 specimens, 1165 (82.9%) were identified as *Anopheles arabiensis* and 241 (17.1%) as *Anopheles gambiae sensu stricto*. Mortality results indicated a significant increase in resistance to lambda-cyhalothrin from 2011 to 2013 in 83% of the sites, permethrin in 25% of the sites, deltamethrin in 25% of the sites and DDT in 50% of the sites. Mosquitoes from 83% of the sites showed full susceptibility to bendiocarb and 17% of sites were suspected to harbour resistance that requires further confirmation. No resistance was observed to fenitrothion in all study sites during the entire survey. The *kdr* genotype results in *An. gambiae s.s.* showed that 67 (50%) possessed susceptibility (SS) alleles, while 35 (26.1%) and 32 (23.9%) mosquitoes had heterozygous (RS) and homozygous (RR) alleles, respectively. Of the 591 *An. arabiensis* genotyped, 425 (71.9%) possessed homozygous (SS) alleles while 158 (26.7%) and 8 (1.4%) had heterozygous (RS) and homozygous (RR) alleles, respectively. Metabolic resistance involving oxidase enzymes was also detected using the synergist PBO.

**Conclusion:**

This is the first nationwide study of insecticide resistance in malaria vectors in Rwanda. It shows the gradual increase of insecticide resistance to pyrethroids (lambda-cyhalothrin, deltamethrin, permethrin) and organochlorines (DDT) and the large presence of target site insensitivity. The results demonstrate the need for Rwanda to expand monitoring for insecticide resistance including further metabolic resistance testing and implement an insecticide resistance management strategy to sustain the gains made in malaria control.

## Background

Most countries in Africa depend heavily on two vector control interventions in their battle against malaria: long-lasting insecticidal nets (LLINs) and indoor residual spraying (IRS). These tools use insecticides from four chemical classes: organochlorines, pyrethroids, carbamates and organophosphates. Whereas 14 formulations belonging to these classes are approved by the World Health Organization (WHO) for use in IRS [1], only pyrethroids are approved for use in LLINs [2] because of their low mammalian toxicity, excito-repellent properties and rapid knock-down and killing effect [3]. It has been estimated that since 2000 more than 670 million cases of malaria have been averted by combining IRS and LLINs with case management and community education [4, 5]. In Rwanda, the national scale-up of vector control interventions has contributed to a steady reduction of malaria cases from 1.6 million in 2005 to 472,000 cases in 2012 [6, 7]. This reduction has been attributed to the combined effects of universal coverage with LLINs [7] and targeted IRS operations in districts with the highest malaria endemicity (5 out of 30 districts) based on epidemiologic and entomologic data. The Rwanda Malaria Indicator Survey carried out in 2013 showed that overall LLIN coverage is high with 83% of the households owning at least one LLIN and 74% reported to have slept under a LLIN the previous night for pregnant women and children under five [8]. From 2005 to 2013, the National Malaria Control Programme (NMCP) of the Rwanda Ministry of Health has distributed approximately 11.2 million LLINs to a population of an estimated 11 million people [9].

From 2007 to 2012, nationwide distributions of LLIN have been conducted in conjunction with annual IRS applications of pyrethroids in high malaria transmission districts either in focal sectors or district-wide by blanket spraying, covering an estimated 98% of the targeted structures. In 2013, the NMCP shifted from pyrethroids to the use of carbamates (Bendiocarb 80% WP) for IRS as part of an insecticide resistance management strategy. This switch was because of confirmed pyrethroid resistance and following the WHO guidance of using active ingredients with different modes of action in rotation [6, 10].

The main mechanisms by which mosquitoes display resistance to insecticides are the expression of elevated levels of detoxifying enzymes (metabolic resistance) and target site insensitivity (knock-down mutations or altered acetylcholinesterase) [10, 11]. Two point mutations in the voltage-gated sodium channel are associated with knock down resistance (*kdr*) to DDT and pyrethroids in the malaria mosquito *Anopheles gambiae s.s.* [12]. One mutation involves a leucine (TTA) to phenylalanine (TTT) substitution at residue 1014 of the gene (L1014F). This mutation is mainly found in West Africa and hence named *kdr*-west [13]. The other mutation involves a leucine (TTA) to serine (TCA) substitution at the same residue (L1014S) and is mostly found in East Africa (*kdr*-east) [14], although both mutations co-occur in some parts of Africa [15].

In 2012, the WHO reported that insecticide resistance in malaria vectors had already been found in more than 64 malaria endemic countries worldwide, with the majority reporting resistance to pyrethroids [10]. This spread is alarming as it poses serious threats to the efficacy of vector control interventions and the gains made in malaria control over the last 10 years. Therefore, it is concerning that most national malaria control programmes (NMCPs) continue to use pyrethroid insecticides for vector control. The situation is also compounded by the extensive use of pyrethroids in agriculture, which poses an additional selection pressure on malaria vectors, for example via insecticide-contaminated ground water that permeates to mosquito larval habitats [14, 16, 17].

The WHO calls for all countries to develop and implement insecticide resistance management strategies in their malaria control programmes in order to curb the spread of resistance as well as preserve the effectiveness of LLINs [10]. Many African countries have now implemented entomological monitoring and susceptibility testing. To inform decisions in the control of malaria in Rwanda, the NMCP has been conducting entomological monitoring of malaria vectors in 12 sentinel sites throughout the country since 2010. Both *An. gambiae s.l.* (94.3%) and *Anopheles funestus* (5%) were the dominant *Anopheles* species. *Anopheles arabiensis* was the predominant sibling species of the *An. gambiae* complex (E.H., unpublished data). Mosquito susceptibility to the WHO recommended classes of insecticides are included annually in this routine survey to monitor resistance to organochlorines, organophosphates, pyrethroids and carbamates [18]. Here, findings of susceptibility tests conducted in 2011 and 2013 to detect knock-down mutations and mortality rates in female *An. gambiae s.l.* mosquitoes are described. This is the first report presenting nationwide results on the composition of the *An. gambiae* complex and its susceptibility to insecticides used for malaria control.

## Methods

### Study area

**Fig. 1:**
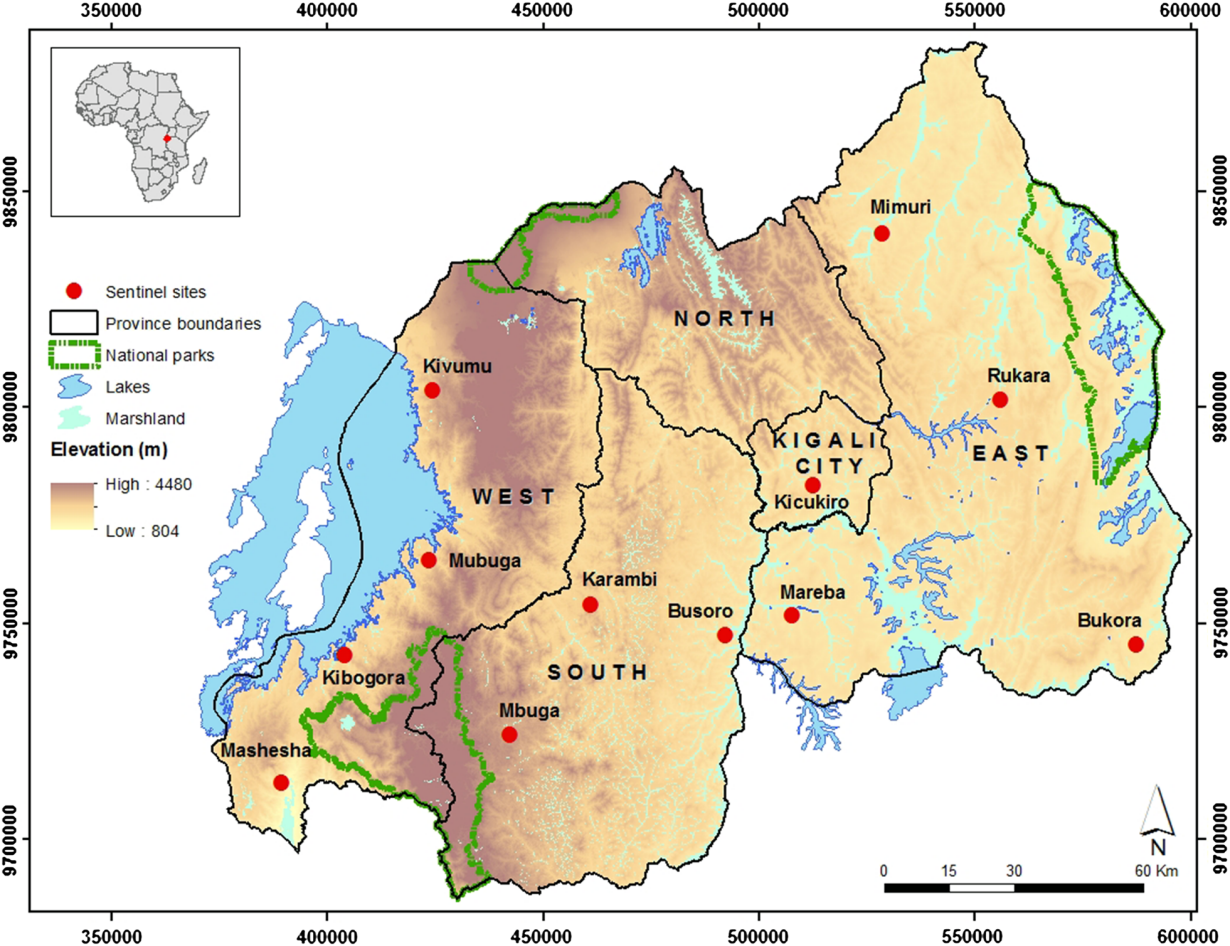
Geographic location of the 12 sentinel sites used for insecticide resistance studies, Rwanda, 2011 and 2013

The study was carried out in 2011 and 2013 in 12 sentinel sites that are distributed over the three provinces of Rwanda (Eastern, Southern and Western province) and Kigali City, all having different levels of malaria endemicity. The 12 sentinel sites are: Busoro, Mbuga and Karambi in Southern Province; Rukara, Mimuri, Bukora and Mareba in Eastern Province; Mashesha, Kivumu, Mubuga and Kibogora in Western Province; and Kicukiro in Kigali City (Fig. 1).

### Mosquito collection

Mosquito larvae were collected in the 12 sentinel sites with standard dippers (350 ml) from stagnant water bodies particularly in rice paddies and other temporal breeding habitats typical for *Anopheles* mosquitoes. All mosquito larvae were collected between 8 and 11 a.m. and brought to a malaria entomology laboratory in the field site where they were reared to adults. Emerging adult *Anopheles* mosquitoes were put in holding cages and fed with 10% sugar solution from cotton wool pads. The adult holding room temperature was between 22 and 28 °C with a relative humidity of 70–80%. Approximately 15,000 *An. gambiae s.l.* mosquitoes were reared to the adult stage, of which 13,807 female mosquitoes were tested for insecticide susceptibility according to WHO protocols [18, 19]. A sample of 10% of these mosquitoes was sent to the International Centre of Insect Physiology and Ecology (*icipe*), Kenya for molecular identification and *kdr* genotyping [20, 21].

### Susceptibility tests

Tests were conducted using kits and insecticide-impregnated filter papers supplied by the WHO collaborating center at the University Sains Malaysia [18]. A batch of 20–25 non blood-fed female mosquitoes that were between 2 and 3 days old were exposed for 1 h to the standard diagnostic concentrations of deltamethrin (0.05%), permethrin (0.75%), lambda-cyhalothrin (0.05%), DDT (4%), bendiocarb (0.1%) and fenitrothion (1%). Each test was run in four replicates and there was one control of 25 field-collected *An. gambiae s.l.* per test. The control mosquitoes were exposed to silicone oil impregnated paper for a similar period. The number of knocked down mosquitoes was recorded at 10, 15, 20, 30, 40, 50 and 60 min [18]. After 60 min, tested mosquitoes were transferred into the holding tube and supplied with 10% sugar solution for 24 h after which the final mortality was scored.

In order to explore metabolic resistance, a pre-exposure of adult female mosquitoes for 1 h to a synergist (piperonyl butoxide: PBO 4%) was carried out according to WHO protocols in six sites where resistance to pyrethroids was confirmed [19]. These tests were only carried out in 2013 and conducted alongside the susceptibility testing with the main insecticides used for malaria control, permethrin 0.75% and deltamethrin 0.05%. After 1 h pre-exposure to PBO, the mosquitoes were transferred to the tubes lined with the corresponding insecticide-impregnated paper. The counting of knockdown and mortality of mosquitoes pre-exposed to PBO was conducted as described.

### Mosquito identification

All mosquitoes were identified to species based on morphological characteristics [22] and stored individually over silica gel awaiting molecular identification and detection of *kdr* mutations. From each sentinel site, 10% of female mosquitoes plus all mosquitoes classified as ‘resistant’ from each site were sent for molecular characterization to the *icipe* laboratory in Kenya where genomic DNA was extracted from the mosquitoes and amplified using specific diagnostic primers for *An. gambiae* [20, 21].

Mutations associated with knock-down resistance (*kdr*) genes were assayed [11] and the DNA products electrophoresed on a 2% agarose gel with ethidium bromide stain and visualized under UV light to identify the presence of susceptible and resistant alleles.

### Interpretation of susceptibility test results

In all tests conducted, the observed mortality in the control tubes was less than 5% and therefore Abott’s correction was not applied [18, 19]. Mortality was calculated as the percentage of individuals that died within 24 h of exposure. Mosquito populations were considered ‘susceptible’ when mortality was between 98 and 100%. When mortality was below 90%, the mosquitoes were classified as ‘resistant’. According to the WHO protocol, an intermediate mortality of 90–97% is suggestive of the existence of resistance and indicates that further investigations are needed [19]. For the prior exposure of mosquitoes to the synergist, a return to full susceptibility to the insecticide in the WHO tube test compared to the WHO tube test with the insecticide-impregnated paper alone indicates that metabolic resistance plays a role in the insecticide resistance observed.

### Statistical analysis

The mortality data were analysed and compared using a Generalized Linear Model with a binomial distribution (IBM SPSS statistics V.20, Chicago, IL, USA). The output provided estimated marginal means of mortality, 95% confidence intervals, standard errors and p values based on Chi square tests.

## Results

### Morphological and molecular identification

A total of 14,311 mosquitoes tested for insecticide resistance was morphologically identified as *An. gambiae s.l.* Of these, 1406 samples collected from 10 sentinel sites were identified by PCR of which 1165 (82.9%) were *An. arabiensis* and 241 (17.1%) *An. gambiae s.s.* (Table 1). Except for Mimuri, *An. arabiensis* was the dominant species in all sentinel sites.

**Table 1:** Species composition of *Anopheles gambiae s.l.* collected in Rwanda, 2011 and 2013 for insecticide resistance tests

Collection site	Total number tested	*An. gambiae s.s.*	*An. arabiensis*
Bukora	200	32 (16%)	168 (84.0%)
Busoro	154	10 (6.5%)	144 (93.5%)
Kicukiro	17	5 (29.4%)	12 (70.6%)
Kivumu	56	3 (5.4%)	53 (94.6%
Mareba	261	16 (6.1)	245 (93.9%)
Mashesha	224	53 (23.7%)	171 (76.3%)
Mimuri	144	89 (61.8%)	55 (38.2%)
Mubuga	183	5 (2.7%)	178 (97.3%)
Kibogora	101	25 (24.8%)	76 (75.2%)
Rukara	66	3 (4.5%)	63 (95.5%)
Total	1406	241 (17.1%)	1165 (82.9%)

### Mortality rates

In 2011, mortality results showed that out of the 12 sentinel sites, mosquitoes were fully susceptible to lambda-cyhalothrin in all sites except for Mashesha, where resistance was potentially emerging with a mortality rate of 97.6%. Mosquitoes were fully susceptible to permethrin in 33% of the sites, were suggested to be resistant in 33% of sites (i.e. had an intermediate mortality of 90–97%) and were classified as resistant in the remaining 34% of sites. The permethrin resistant populations were from Bukora (84% mortality), Kibogora (89% mortality), Mimuri (86% mortality) and Rukara (84% mortality). For deltamethrin, mosquitoes were fully susceptible in 58% of sites. In 33% of sites, the existence of resistance was suggested. Resistance to deltamethrin was confirmed for one site: Bukora (85% mortality). For DDT, 28% of sites showed full susceptibility, 36% of sites had a suspicion of resistance and resistance was confirmed in another 36% of sites surveyed: Bukora (77% mortality), Kibogora (75% mortality), Kicukiro (52% mortality) and Mashesha (88% mortality). Mosquitoes were fully susceptible to bendiocarb in 92% of sites. In one site (Kibogora), the mortality of 96% is suggestive of the existence of resistance to bendiocarb. Fenitrothion was the only insecticide to which mosquitoes showed full susceptibility in all the sites tested with 100% mortality after 24 h (Table 2).

**Table 2:** Mortality rates of female *Anopheles gambiae s.l.* tested for insecticide resistance to six insecticides in 2011 and 2013 in 12 sentinel sites in Rwanda

Insecticide	2011				2013				
	Site	N	# Replicates	Mortality (%)	N	# Replicates	Mortality (%)	χ^2^ value	p value
Lambdacyhalothrin 0.05%	Bukora	86	4	99 ± 1	100	4	63 ± 5	51.3	<0.001*
	Busoro	82	4	100	200	8	72 ± 3	77.78	<0.001*
	Karambi	84	4	100	99	4	93 ± 3	7.53	0.006*
	Kibogora	81	4	99 ± 1	100	4	85 ± 4	13.29	<0.001*
	Kicukiro	82	4	100	191	8	75 ± 3	64.112	<0.001*
	Kivumu	82	4	100	100	4	100		
	Mareba	88	4	100	100	4	43 ± 5	132.1	<0.001*
	Mashesha	92	4	98 ± 1	94	4	87 ± 3	8.37	0.04*
	Mbuga	81	4	100	97	4	95 ± 2	5.27	0.02*
	Mimuri	85	4	100	100	4	57 ± 5	75.44	<0.001*
	Mubuga	83	4	100	85	4	98 ± 2	2.05	0.15
	Rukara	82	4	100	100	4	78 ± 4	28.02	0.000*
Permethrin 0.75%	Bukora	85	4	84 ± 4	200	8	84 ± 3	0.1	0.92
	Busoro	100	4	100	100	4	95 ± 2	5.26	0.02*
	Karambi	88	4	91 ± 3	99	4	91 ± 3	0	0.98
	Kibogora	84	4	89 ± 3	100	4	92 ± 3	0.367	0.54
	Kicukiro	83	4	99 ± 3	97	4	97 ± 2	0.772	0.38
	Kivumu	80	4	100	100	4	100		
	Mareba	92	4	99	100	4	63 ± 5	53.9	<0.001*
	Mashesha	183	8	96 ± 3	94	4	90 ± 3	3.04	0.08
	Mbuga	87	4	95 ± 3	97	4	100		
	Mimuri	87	4	86 ± 4	100	4	66 ± 5	11.03	<0.001*
	Mubuga	90	4	97 ± 2	85	4	94 ± 3	0.72	0.39
	Rukara	86	4	84 ± 5	100	4	92 ± 3	2.95	0.09
Deltamethrin 0.05%	Bukora	168	8	85 ± 3	97	4	74 ± 4	3.85	0.05
	Busoro	84	4	100	200	8	88 ± 2	28.57	<0.001*
	Karambi	82	4	99 ± 1	100	4	97 ± 1	0.72	0.4
	Kibogora	83	4	93 ± 3	97	4	89 ± 3	0.92	0.34
	Kicukiro	86	4	90 ± 3	194	4	82 ± 3	3.1	0.08
	Kivumu	85	4	100	100	4	100		
	Mareba	97	4	99	100	4	67 ± 5	45.1	<0.001*
	Mashesha	99	4	99 ± 2	95	4	97 ± 2	1.22	0.27
	Mbuga	89	4	97 ± 2	86	4	100	2.05	0.15
	Mimuri	86	4	98 ± 2	100	4	81 ± 4	15.42	<0.001*
	Mubuga	90	4	99	90	4	98 ± 2	1.34	0.56
	Rukara	87	4	94.5 ± 2	100	4	90 ± 2	1.37	0.24
DDT 4%	Bukora	86	4	77 ± 5	94	n/a	n/a		
	Busoro	84	4	100	100	4	89 ± 3	12.36	<0.001*
	Karambi	86	4	95 ± 2	95	4	87 ± 3	3.8	0.05*
	Kibogora	84	4	75 ± 2	0	4	n/a		
	Kicukiro	83	4	52 ± 6	93	4	43 ± 5	1.37	0.242
	Kivumu	88	4	95 ± 2	100	4	100	4.2	0.04*
	Mareba	88	4	99	100	4	81 ± 4	19.9	<0.001*
	Mashesha	94	4	88 ± 3	99	4	70 ± 3	14.07	0.001*
	Mbuga	83	4	100	84	4	96 ± 2	3.11	0.08
	Mimuri	85	4	95 ± 2	100	4	76 ± 5	13.22	<0.001*
	Mubuga	90	4	96 ± 2	89	4	96 ± 2	0.000	0.99
	Rukara	n/a	n/a	n/a	100	4	94 ± 2		
Bendiocarb 0.1%	Bukora	87	4	100	100	4	91 ± 3	8.81	0.003*
	Busoro	88	4	100	100	4	100		
	Karambi	84	4	99 ± 1	83	4	98 ± 1	0.202	0.65
	Kibogora	83	4	96 ± 2	97	4	100	3.11	0.08
	Kicukiro	93	4	100	96	4	100		
	Kivumu	82	4	100	100	4	96 ± 2	3.1	0.08
	Mareba	87	4	100	100	4	100		
	Mashesha	93	4	100	100	4	100		
	Mbuga	86	4	99	88	4	100	1.01	0.31
	Mimuri	83	4	100	88	4	100		
	Mubuga	91	4	100	81	4	100		
	Rukara	86	4	99	96	4	100		
Fenitrothion 1%	Bukora	83	4	100	97	4	100		
	Busoro	93	4	100	100	4	100		
	Karambi	82	4	100	94	4	100		
	Kibogora	81	4	n/a	90	4	100		
	Kicukiro	85	4	100	98	4	100		
	Kivumu	84	4	100	100	4	100		
	Mareba	84	4	100	100	4	100		
	Mashesha	183	8	100	100	4	100		
	Mbuga	81	4	100	192	8	100		
	Mimuri	82	4	100	88	4	100		
	Mubuga	82	4	100	88	4	100		
	Rukara	82	4	100	100	4	100		

Two years later, in 2013, susceptibility to lambda-cyhalothrin was recorded in one site only, while recorded mortalities in 25% of sites were suggestive of the existence of resistance. Resistance was confirmed in 66% of sites. *Anopheles gambiae s.l.* was resistant to permethrin in 25% of sites, showed susceptibility to permethrin in 16% of sites, and mortalities from 58% of the sites were suggestive of the existence of resistance. For deltamethrin, the mosquitoes showed susceptibility in 25% of the sites, a suggestion of the existence of resistance in 25% of sites, and full resistance in 50% of sites. The test of resistance to DDT was carried out in 10 sites only, and susceptibility was recorded in 10% of sites, whereas 30% of sites suggested the existence of resistance, and full resistance was observed in 60% of sites. Mosquitoes were fully susceptible to bendiocarb in 83% of sites, and two sites (Bukora and Kivumu) suggested the existence of resistance. Mosquitoes were susceptible to fenitrothion in all sites.

Comparing the mortality rates of 2011 and 2013, there was a significant increase in resistance levels to lambda-cyhalothrin in 83% of the sites, to permethrin in 25% of sites, to deltamethrin in 25% of sites and to DDT in 50% of sites. A significant decrease of susceptibility of *An. gambiae s.l.* to bendiocarb was found in one site (Bukora) and, as in 2011, all mosquitoes tested remained 100% susceptible to fenitrothion in 2013 (Table 2).

### *kdr* Genotypes

Seven hundred and twenty-five (134 *An. gambiae s.s.* and 591 *An. arabiensis*) mosquitoes collected in 2011 and 2013 were genotyped for *kdr*-east (L1014S). Of the 134 *An. gambiae s.s.* genotyped, 67 (50%) possessed susceptibility (SS) alleles, while 35 (26.1%) and 32 (23.9%) had heterozygous (RS) and homozygous (RR) alleles, respectively. Of the 591 *An. arabiensis* that were genotyped, 425 (71.9%) possessed homozygous (SS) alleles, while 158 (26.7%) and 8 (1.4%) had heterozygous (RS) and homozygous (RR) alleles, respectively. *Anopheles gambiae s.s.* and *An. arabiensis* from Mimuri (Eastern Province) contributed disproportionately to the total observed frequency of the homozygous resistance genotype (RR), with 36 and 12% for the two species, respectively (Table 3).

**Table 3:** Variation of *kdr* genotypes (RR, RS and SS) in *An. gambiae s.s.* and *An. arabiensis* collected from seven sentinel sites in 2011 and 2013

Site	Total tested	*An gambiae s.s.* genotype count (allele frequency)				*An. arabiensis* genotype count (allele frequency)			
		N	SS (%)	RS (%)	RR (%)	N	SS (%)	RS (%)	RR (%)
Busoro	44	4	0 (0.00)	4 (1.00)	0 (0.00)	0	12 (0.30)	28 (0.70)	0 (0.00)
Kicukiro	17	5	0 (0.00)	5 (1.00)	0 (0.00)	12	0 (0.00)	12 (1.00)	0 (0.00)
Kivumu	56	3	3 (1.00)	0 (0.00)	0 (0.00)	53	53 (1.00)	0 (0.00)	0 (0.00)
Mareba	256	17	16 (0.94)	1 (0.06)	0 (0.00)	239	226 (0.96)	13 (0.04)	0 (0.00)
Mashesha	24	12	0 (0.00)	12 (1.00)	0 (0.00)	12	1 (0.08)	11 (0.92)	0 (0.00)
Mimuri	145	88	43 (0.49)	13 (0.15)	32 (0.36)	57	49 (0.86)	1 (0.02)	7 (0.12)
Mubuga	183	5	5 (1.00)	0 (0.00)	0 (0.00)	178	84 (0.47)	93 (0.52)	1 (0.01)
Total	725	134	67 (50.0)	35 (26.1)	32 (23.9)	591	425 (71.9)	158 (26.7)	8 (1.4)

RR denotes the homozygous resistant L1014S genotype and SS denotes the homozygous susceptible wild genotype. The *An. gambiae s.s.* and *An. arabiensis* genotypes count for the 2 years of study were pooled

### Pre-exposure of *Anopheles gambiae s.l.* to piperonyl butoxide

The pre-exposure of *Anopheles gambiae s.l.* to the synergist piperonyl butoxide (PBO) was conducted in six sites where resistance to pyrethroids was confirmed. The test was conducted only with permethrin 0.75% and deltamethrin 0.05%. In all sites, susceptibility was restored and ranged from 98 to 100% (Tables 4, 5).

**Table 4:** Comparison of mortality rates of *Anopheles gambiae s.l.* exposed to permethrin 0.75% alone and permethrin 0.75% + piperonyl butoxide (PBO) per site in 2013

Sites	Permethrin 0.75%		Permethrin 0.75% + PBO		Chi square		
	Total tested	Mortality (%)	Total tested	Mortality (%)	χ^2^	*df*	p
Bukora	200	84 ± 3	100	98 ± 2	9.4	1	0.02
Kibogora	100	92 ± 3	100	99 ± 1	5.9	1	0.015
Kicukiro	97	97 ± 2	100	100	3.1	1	0.08
Mareba	100	63 ± 5	100	100	58.7	1	<0.001
Mimuri	100	66 ± 5	100	98 ± 2	41.9	1	<0.001
Rukara	100	75 ± 5	100	100	32	1	<0.001

**Table 5:** Comparison of mortality rates of *Anopheles gambiae s.l.* exposed to deltamethrin 0.05% alone and deltamethrin 0.05% + piperonyl butoxide (PBO) per site in 2013

Sites	Deltamethrin 0.05%		Deltamethrin 0.05% + PBO		Chi square		
	Total tested	Mortality (%)	Total tested	Mortality (%)	χ^2^	*df*	p
Bukora	97	89 ± 3	100	100	12.4	1	<0.001
Kibogora	97	89 ± 3	100	100	12.4	1	<0.001
Kicukiro	194	84 ± 4	98	100	17.8	1	<0.001
Mareba	100	67 ± 5	100	100	49.2	1	<0.001
Mimuri	100	81 ± 4	100	98 ± 2	13.9	1	<0.001
Rukara	100	84 ± 4	100	100	19	1	<0.001

## Discussion

In Rwanda, integrated malaria control interventions (artemisinin-based combination therapy (ACT), LLINs and targeted IRS) have been in use since 2006. This has contributed to a significant reduction in clinical malaria cases in the country [7]. However, the gains made are fragile due to the decrease of efficacy of interventions, partially as a result of insecticide resistance development that has spread throughout Africa [4, 23, 24]. Rwanda achieved universal coverage with LLINs in 2011, but the major challenge is to maintain this coverage and use with effective mosquito nets, especially after it was recently reported for Rwanda that LLIN effectiveness lasts less than 3 years due to the rapid loss of insecticidal activity and physical deterioration in the field [25]. LLIN deterioration problems were also shown in recent findings from Senegal where damaged nets provided less protection from malaria compared to intact ones [26].

The fact that millions of nets may have lost their effectiveness and thus continue to expose mosquitoes to a sub-lethal dose of pyrethroids is of major concern, because this may contribute to the further development of resistance. Similarly, annual consecutive use of pyrethroids in IRS, combined with extensive use of pyrethroids in agriculture has also implications for emerging insecticide resistance [27]. Hence, Rwanda with the support of PMI and the Global Fund has been keen to monitor insecticide resistance so that it can take action to mitigate the emergence of resistance. In 2010, an initial susceptibility survey was conducted in eight sites in which mosquitoes were tested with deltamethrin, permethrin, bendiocarb, malathion and DDT using the CDC bottle assay [28, 29]. These mosquitoes were found to be fully susceptible, except to DDT in two sites (E.H., unpublished data).

The data collected in the current surveys (2011 and 2013) confirm that resistance to DDT has been on the rise since 2011. In addition, the results show resistance to the pyrethroids (deltamethrin, permethrin, and lambda-cyhalothrin) being present in 2011 and further increasing in 2013. Although DDT was banned for usage in Rwanda in 1989, high levels of resistance are of concern because resistance to DDT confers cross-resistance to pyrethroids [10]. Mosquitoes were fully susceptible to fenitrothion in all the sites, while possible emergence of resistance to bendiocarb was reported in the present study. Nevertheless, a switch to IRS with bendiocarb was made from September 2013 onwards so that the two main vector control interventions (LLINs and IRS) employed different classes of insecticide to delay resistance development.

The presence of *kdr* mutations recorded in some sites can be explained by intense indoor interventions with IRS and LLINs, as was reported in Burundi and Tanzania [30, 31]. However, it should be noted that the frequency of *kdr* in the vector population may not be a reliable marker for actual resistance [32] and that care should be taken when interpreting such data as other resistance mechanisms can play a role as well. In current study, metabolic resistance involving oxidases was proven by using piperonyl butoxide (PBO). Susceptibility was fully restored in the six selected sites where resistance to pyrethroids had been identified. This suggests that metabolic resistance may even account for all observed resistance in bioassays with malaria vectors from Rwanda.

The resistance level in Mimuri (Nyagatare District) could explain the high number of malaria cases occurring in this district: about 42% of all malaria cases of the 30 districts in 2011 were found here [33] before the introduction of bendiocarb for IRS. This site, as well as Mashesha, Mareba, and Kibogora, is characterized by rice growing in which agricultural pesticides, and pyrethroids in particular, are extensively used.

In response to these findings, Rwanda developed and implemented an insecticide resistance management (IRM) plan in 2013 that recommended transitioning to non-pyrethroid IRS to mitigate pyrethroid resistance and to lengthen the effectiveness of LLINs [6]. Rwanda transitioned to carbamate (bendiocarb) for IRS and the country will switch to a long-lasting organophosphate (Actellic–pirimiphos-methyl CS) in 2016 due to the reported trends of suggestive resistance to bendiocarb. The question remains, however, whether Rwanda should continue with blanket IRS treatment in an entire district when data on resistance is reported from only one sentinel site per district. To answer this question, it is recommended that additional sites should be identified in the targeted districts in order to determine whether or not the spread of resistance is homogeneous throughout the district. Similar to a recent report from Malawi, the use of long-lasting IRS formulations, such as pirimiphos-methyl, may be costly initially, but cost-effective in the longer term in managing insecticide resistance [34].

In this study, characterization of *An. gambiae s.l.* from 10 sentinel sites revealed that the predominant sibling species is *An. arabiensis* (83%). This is contrary to a study conducted in one site near Kigali City in 2007 by PMI-Rwanda, in which it was reported that *An. gambiae s.s.* accounted for 93.6% of the total 157 *An. gambiae s.l.* examined by PCR while *An. arabiensis* accounted for only 6.4% [35]. Although the earlier sampling was carried out in one site only, the results suggest that *An. gambiae s.s.* was the predominant species before the scale-up of interventions with LLINs and IRS. Such a shift in species composition has been reported in neighbouring countries, for instance in Kenya, Uganda and Tanzania [36–38]. This phenomenon has important implications for malaria epidemiology and control given that *An. arabiensis* is an opportunistic feeder which has a tendency to rest and feed on humans outdoors [39]. Outdoor-biting mosquitoes are less susceptible to indoor interventions and therefore outdoor interventions that supplement LLINs and IRS will need to be instituted in the context of an integrated approach to vector management [38, 40]. Although *An. arabiensis* is the dominant vector in Rwanda and resistance levels are high in *An. gambiae s.l.* populations, future studies should include testing of resistance by sibling species to assess differences in susceptibility levels.

With the goal of reaching the pre-elimination phase of malaria by 2018, the Ministry of Health developed an integrated vector management (IVM) strategy which aims at improving the efficiency, effectiveness, ecological soundness and sustainability of vector control interventions in Rwanda [41]. Entomological monitoring, including testing for insecticide resistance, is now a major part of the NMCP in Rwanda and a rotation strategy for management of insecticide resistance has been adopted in line with IVM.

Meanwhile, with spreading insecticide resistance and behavioural change of malaria vectors, there is an interest to integrate other innovative interventions which do not rely on insecticides [42, 43]. Larval control interventions have proven cost-effective across a range of different settings and include application of environmental management, insect growth regulators, and biological control [44]. Recently, successful field experiments have been carried out with microbial larvicides in Tanzania, Kenya, the Gambia and Benin. These showed a substantial impact on malaria disease [45–47]. It was demonstrated that for a sustainable solution, a horizontally organized community-based programme that takes the needs and wishes of people into account has to be established and technically empowered [48, 49]. Currently, Rwanda is supporting a research project in Ruhuha (South East Rwanda) that aims to involve communities in the application of the microbial larvicide *Bacillus thuringiensis* var. *israelensis* (Bti) (E.H., unpublished data). This may form the basis for the integration of alternative vector control interventions for the management of insecticide resistance [42].

## Conclusion

The results of the study show that resistance to pyrethroids and organochlorines (DDT) was documented in 2011 and has been on the rise between 2011 and 2013. An emerging resistance to carbamates (bendiocarb) was found in few sites and gives reasons for concern of its efficacy in the near future. No resistance was recorded for organophosphates (fenitrothion). Probably due to the use of indoor insecticide-based interventions, the more opportunistic and outdoor feeding *An. arabiensis* was the dominant sibling species in the country. To curb further spread of pyrethroid resistance and to preserve the effectiveness of LLINs, Rwanda implemented an insecticide resistance management strategy in 2013 and switched to carbamates (bendiocarb) for IRS. It plans to implement a rotational strategy of insecticides, including organophosphates, every 2–3 years. The results reported here are the first results on the status of insecticide resistance in Rwanda and indicate that the NMCP should continue monitoring insecticide resistance for target site insensitivity and metabolic resistance so as to guide future decisions on insecticide use for public health.

## Abbreviations

ACT: artemisinin-based combination therapy
*Bti*: *Bacillus thuringiensis* var. *israelensis*
CDC: Centers for Disease Control and Prevention
DDT: dichlorodiphenyltrichloroethane
*kdr*: knockdown resistance
LLIN: long-lasting insecticide-treated net
IRS: indoor residual spraying
IVM: integrated vector management
NMCP: National Malaria Control Programme
PBO: piperonyl butoxide
PCR: polymerase chain reaction
PMI: President’s Malaria Initiative
WHO: World Health Organization
